# Survey on the tourist satisfaction of rural homestay based on the theory of importance–performance analysis

**DOI:** 10.3389/fpsyg.2022.940345

**Published:** 2022-08-11

**Authors:** Xiaorui Zhou, Yanan Wang, Pingping Ma

**Affiliations:** Faculty of Business, Guilin University of Technology, Guilin, China

**Keywords:** IPA analysis, rural homestay, tourist satisfaction, network text analysis, emotional change

## Abstract

Guilin rural homestays are an important support for rural tourism destinations, serving not only as accommodation but also as a representative of the local culture of the town. To improve satisfaction with rural homestays among tourists, enhance destination attractiveness, and better meet tourist demands for accommodation conditions, this study combines literature and network text analysis to construct an evaluation index system for Guilin rural homestay tourist satisfaction. The data collected by a questionnaire survey based on importance–performance analysis (IPA) are analyzed. The results show that actual tourist satisfaction with the experience in the 21 indexes is lower than the pre-consumption expectation, due to the imperfect facilities, lack of special service development, relative optimization of basic road construction, and the need for improvement in the internal and external environment, among other factors. Through the improvement of the above factors, the satisfaction of tourists to the rural homestay can be improved.

## Introduction

As an emerging representative of the homestay industry, rural homestays are an important support for rural tourism. They make full use of idle old houses and land resources in the countryside, as well as employ the rural population, thus bringing vitality and vigor to the countryside and helping the development of the rural economy. Rural residential accommodation is an important part of rural tourism planning. Subsidized by relevant policies, idle rural land resources are being built and improved to provide rural tourism consumers with a return to nature—a mind–body retreat. Tourist satisfaction plays an important role in the success of tourist destinations (Meng et al., 2006; Yan, 2018). By studying the degree of satisfaction with rural homestays among tourists, this study has an important theoretical basis and is of practical significance for the industry’s sustainable future development.

The term “homestay” originated in Japan and refers to small self-employed hotels that provide a non-commercial, home-like environment, as well as breakfast (Nuntsu et al., 2004; Chen et al., 2013; Yang, 2016). According to the Chinese Ministry of Culture and Tourism, a homestay refers to the use of idle resources, such as local residences, to operate short-term occupancy accommodations with no more than four floors, and a construction area of no more than 800 square meters; the host’s participation in the reception provides visitors with an experience of the local natural, cultural, and productive lifestyle in small accommodation facilities (LB/t065-2019, 2019). According to the Organization for Economic Cooperation and Development, rural tourism is defined as tourism that takes place in the countryside: “Villages are the focus and unique selling point of the rural tourism package” (Reichel et al., 2000). With the development of tourism, the homestay industry has become a unique and rapidly developing sector in the hotel industry (Chiu, 2018). The form of homestays began to diversify, as a new form of homestay, the village homestay (which makes use of idle houses), appeared widely in scenic tourist destinations. According to the existing literature, the rural residential accommodations in this study refer to places where the villagers make full use of their own idle houses, creating a tourist offer that combines the local regional culture, human environment, natural landscape, production, and living forms while providing tourists with the experience of rural residential services.

Tourist satisfaction is an important aspect of the tourism service industry (Kozak et al., 2004). In a study of the factors influencing satisfaction with homestay accommodations, Stringer (1981) has examined the preferences of Australian tourists traveling to the United Kingdom and found that the tourists’ usual consumer preferences, the location of the accommodation, the environment of the tourist’s place of residence, and their understanding of the destination have a great influence on satisfaction. According to the customer satisfaction system constructed by Zhao and Wu (2013) for the Beijing Economy Hotel, the customer-perceived value of an economy hotel is divided into three parts, related to the product, service, and emotional values. Jiang and Sheng (2020) collated and analyzed data from a customer satisfaction questionnaire for residential accommodation, with evaluation indexes for consumers related to the external environment, building layout, space layout, service facilities, service quality, and management benefit of a Suzhou rural residential quarters. Chen’s (2018) research and analysis of the customer satisfaction index based on the perceived value and behavioral intention of the hotel based on online comments divides the customer perception of the homestay into five dimensions, related to functional, interactive, affective, cognitive, and social value. Hu (2021) included five categories in an evaluation index system for guest satisfaction with guesthouses in ethnic areas: location, facilities, services, image, and price. Dong et al. (2021) used the grounded theory to construct an influence model for the degree of satisfaction with residential accommodation and concluded that the factors influencing homestay satisfaction fall into six categories: service facilities, geographic location, house facilities, hygiene, environment, and charm points. In view of this, the introduction of the importance performance analysis (IPA) method into the study of tourist satisfaction with rural homestays can better find problems that can be targeted for solutions.

This study analyzes the gap between the products and services provided by rural homestays in tourist destinations in terms of consumer expectations by constructing a tourist satisfaction evaluation index system and analyzing the data using IPA analysis, pointing out the weak factors among them, which have important practical significance for the sustainable development of homestays, and analyzing them in depth to recognize the shortage of homestay facilities and environment, the lack of homestay special services, and the optimization of homestay basic road construction The four aspects of the problems of the homestay, the lack of quality of the homestay operators, as well as providing improvement paths for the homestay and putting forward corresponding suggestions and countermeasures to improve the shortcomings of rural homestay and increase the satisfaction of rural homestay tourists in order to achieve the purpose of increasing the income of rural tourism homestay Studying homestays, exploring the factors and countermeasures affecting their tourism attractiveness, the same has important theoretical significance for the sustainable development of homestays, and gaining an in-depth understanding of the connotation of the perceived value of rural homestays. This paper hopes to enrich and extend the research boundary of tourists’ perceived value through the study of the perceived value of homestays and inspire and attract more scholars to focus on and study the perceived value of rural homestays. It also systematically analyzes the difference between the actual satisfaction and importance of perceived value of rural homestay. At present, there are few studies on the perceived value of rural homestay tourists using IPA analysis, and the research on the perceived value of rural homestay using IPA analysis has not been systematically conducted. This study uses IPA analysis to provide a new research method for the study of the perceived value of homestay visitors.

## Research design

### Methodology

#### Importance–performance analysis

Importance–performance analysis is also known as importance–satisfaction analysis in China. At first, IPA was mainly used in the research of image perception, but as the method gradually became more widely known, the scope of its use also became more widespread in the analysis of, for example, service quality, destination image, market segmentation, and destination competitiveness (Ennew et al., 1993; Ford et al., 1999; Baloglu and Mangaloglu, 2001; Wade and Eagles, 2003; Croes and Kubickova, 2013). IPA, formally proposed by Martilla and James (1977), has been applied to the research of various industries, especially the service industry, with importance (expectation) listed as the vertical axis, and performance (satisfaction) as the horizontal axis (Martilla and James, 1977). American researcher Evans and Chon (1989) was the first to apply IPA to the study of satisfaction in the context of travel destinations (Evans and Chon, 1989). Oh and Parks (1998) conducted an empirical study on customer satisfaction and tourism using the IPA method. Domestic research on the IPA method has involved various fields in the service industry. Wang and Chen (2017) put forward recommendations for Xiamen Smart Tourism based on data results obtained through the IPA analysis of the four dimensions of facility, service, safety, and environmental experience. Sang et al. (2018) found that Hubei Enshi homestay tourism needed to be improvement in three aspects *via* IPA: the surrounding environment of the homestay, the interaction between the homestay owner and tourists, and the scope of the homestay “home.”

In IPA, the overall and performance averages of the customer product/service attributes are divided into four quadrants. Higher satisfaction and expectation in the first quadrant constitute the superior region, and lower satisfaction and higher expectation in the second quadrant are called the improvement region. Lower expectation in the third quadrant with lower satisfaction is called the opportunity region; while lower expectation with higher satisfaction in the fourth quadrant is called the maintenance region. The IPA method establishes these cross coordinates after inputting the data, and the analysis elements will be directly distributed in each quadrant area so the results are clear and easy to understand. This advantage can help researchers determine the essential factors that need to be improved quickly and effectively. The research on IPA at home and abroad has thus been involved in various fields of the service industry.

#### Web text analysis

Web text analysis extracts a large number of web-based reviews from relevant websites *via* a web crawler and quantitatively analyzes the comment information (Lu and Li, 2020). With the development of online travel booking platforms, more and more people are choosing to book homestays online, and consumers tend to evaluate the homestay experience online as well. A large number of online reviews can have high credibility and scientificity, which greatly influences the homestay choice (Lin et al., 2021). Network text analysis requires segmentation of the text documents related to the reviews, filtering of the words unrelated to the research content, and then analysis of the documents after the segmentation to yield a vocabulary frequency table based on how often a word occurs, from high to low, to select the content for analysis (Zhao and Wu, 2013).

### Construction of evaluation index system

#### Establishment of the high-frequency feature vocabulary

The evaluation index of perceived value comes from Airbnb, Ctrip, Qunar, Meituan, and other OTA online travel booking platforms. To ensure the accuracy and validity of the perceived value system constructed by the keywords extracted from the online reviews, corresponding conditions were set up for data collection: first, the collection areas for online reviews were the three provinces of southwest Yunnan, Guangxi, and Guizhou; second, the score for the homestay had to be 4.5 and above. Octopus data grabber was used to capture the keywords from 1 million online reviews collected from the main domestic online tourism platforms such as Ctrip, Qunar, eLong, Mafengwo, and Tuniu. Table 1 is a summary table of the top 50 keywords in online reviews.

**TABLE 1:** Online rating of high-frequency feature words.

	Characteristic word	Frequency	Part of speech		Characteristic word	Frequency	Part of speech
1	Boss	235577	Nouns	26	Thoughtful	38452	Adjectives
2	Service	208863	Nouns	27	Yard	38259	Nouns
3	Room	192296	Nouns	28	Traffic	37415	Nouns
4	Environment	190514	Nouns	29	Tidy	35846	Adjectives
5	Clean	160536	Adjectives	30	Pretty	33727	Adjectives
6	Inns	153447	Nouns	31	Reception	32619	Nouns
7	Passion	149469	Adjectives	32	Delicious	27568	Adjectives
8	Convenient	125606	Adjectives	33	Geography	27035	Nouns
9	Position	121276	Nouns	34	Housekeeper	26202	Nouns
10	Comfortable	89277	Adjectives	35	Luggage	25086	Nouns
11	Facilities	82643	Nouns	36	Style	24451	Nouns
12	Sanitation	82459	Nouns	37	Arrangement	23182	Nouns
13	Quiet	71781	Adjectives	38	Ancient town	20723	Nouns
14	Check in	68050	Verbs	39	Lay out	15911	Nouns
15	Satisfy	56703	Adjectives	40	Store	19101	Nouns
16	Cozy	53931	Adjectives	41	Supplies	18951	Nouns
17	Attitude	53328	Nouns	42	Hot water	17738	Nouns
18	Friend	49038	Nouns	43	Affordable	17689	Adjectives
19	Warm	46469	Adjectives	44	Intimate	17511	Adjectives
20	Boss lady	45748	Nouns	45	Bathroom	17438	Nouns
21	Furnish	44807	Nouns	46	Experience	17417	Verbs
22	Value for money	43749	Nouns	47	Stay	17364	Verbs
23	Breakfast	43600	Nouns	48	Suggest	17030	Nouns
24	Free	39221	Nouns	49	Pick up	16655	Verbs
25	Features	39098	Nouns	50	Easy to find	16125	Verbs

#### Construction of evaluation index system for satisfaction of rural homestay tourists

Woodruff (1997) believed that the main source of consumer expectation in buying and using goods was the value of the goods themselves, and the degree of satisfaction is determined by whether the actual effect of the commodity itself on the consumer is greater than the expected value the consumer perceived before using the commodity, based on his theory, Woodruff distinguished between perceived and added value. Sheth et al. proposed that any commodity service can be divided into five dimensions of product value: function, society, cognition, emotion, and context. The functional value refers to the value attached to the commodity itself; the emotional value refers to the change in the value of the commodity brought about by the change in consumers’ emotions; and the cognitive value refers to the injection of new knowledge, the new change in the consumer’s cognition, and the new cognition value of goods and services can also be interpreted as their novelty. The social value is the connection between goods and services and social groups, while the situational value refers to the difference in commodity value due to differences and changes in actual situations (Fang and Qu, 2018).

Based on a literature review and analysis, we divide customers’ perceived value in the customer satisfaction system into functional value, homestay service value, the four dimensions of emotional value, and price cognitive value (Xiao, 1996; Tian and Wu, 2011; Wei, 2011; Li, 2012; Zhao and Wu, 2013; Chiao, 2014; Chen, 2018; Fang and Qu, 2018; Yan, 2018; Wu et al., 2018; Jiang and Sheng, 2020; Dong et al., 2021; Hu, 2021). Functional value refers to the basic facilities and equipment of the homestay, such as the road, landmarks, room, bed, and toilet, as well as whether the host is hospitable or not, and whether the staff efficiency is high or low. The emotional value is whether or not the host provides warm-hearted shuttle service, the service of the staff is considerate, and the host prepares local specialties to serve the guests. The cognitive value refers to the evaluation of the cost-effectiveness of the homestay after the tourist experience, and so on.

By clearly summing up the characteristic categories of high-frequency words in the tourists’ online comments, we constructed an evaluation system of perceived value from the perspective of the tourists’ online comments, as shown in Table 2.

**TABLE 2:** Evaluation index for tourist satisfaction with Guilin rural homestay house.

Primary index	Secondary index
A. Functional value satisfaction	A1. External traffic in the homestay
	A2. The homestay is close to the scenic spot
	A3. The homestay room is large
	A4. Room decoration style
	A5. The sanitary condition of the bed and breakfast room is good
	A6. The bed is comfortable
	A7. The bathroom is clean and tidy
	A8. The homestay hot water supply stable
	A9. The room air-conditioning is in normal use and no noise
	A10. The Wi-Fi in the room is fast
	A11. The room has a balcony
	A12. The room is well-insulated
	A13. Courtyard environment
	A14. Overall residential decoration style
B. Satisfaction with the value of bed and breakfast service	B1. Normal charges for bed and breakfast service
	B2. The service attitude of the accommodation staff (warm, considerate, and patient)
	B3. Room service for bed and breakfast (thoughtful, fast)
C. Emotional value satisfaction	C1. Homestay hosts provide guided tour services (professional, detailed, efficient, and considerate)
	C2. The homestay offers local food
	C3. Homestay provides shuttle service, pick up, and drop off station
D. Price perception, value satisfaction	D1. Homestay is cost effective

### Questionnaire design

The questionnaire was divided into three parts. The first part was about the importance of each index and the evaluation of the degree of satisfaction. This section contained 21 indicators, each of which needed to be judged in terms of pre-consumption expectations and post-consumption actual satisfaction, each measured on a 5-point Likert scale, where pre-consumption expectation ranged from 1, not important at all, to 5, very important, and actual experience satisfaction ranged from 1, not at all satisfied, to 5, very satisfied (Wang et al., 2012). The second part contained questions about the basic situation of the homestay, including the number of times, days, access to information, and so on. The third part dealt with demographic characteristics, including gender, age, occupation, monthly income (cost of living), and questions about the provinces. According to the perceived value system of the above collation—and combined with the actual situation of the keyword design questions—the question design is shown in Table 3.

**TABLE 3:** Summary of questionnaire items.

Number of questions	Content of questions
Q1	External traffic of homestay
Q2	Homestay is close to the scenic spot
Q3	The homestay room is large
Q4	The decoration style of the homestay room
Q5	Sanitary conditions of bed and breakfast rooms
Q6	The bathroom is clean and tidy
Q7	The comfort of the bed
Q8	Homestay hot water supply is stable
Q9	Normal use of room air conditioning
Q10	The Wi-Fi in the room is fast
Q11	The room has a balcony
Q12	The room is well-insulated
Q13	There is a courtyard downstairs where you can play
Q14	The overall decoration style of the homestay
Q15	Normal charges for homestay service
Q16	The homestay service staff’s attitude (warm, considerate, patient)
Q17	The homestay room service is very thoughtful and fast
Q18	The host provides the warm service as well as the personalized tour guide service
Q19	Local food is available at the hostel
Q20	Provides pick-up and drop-off car and/or pick-up and drop-off station services
Q21	The homestay price is economical and practical

### Questionnaire

The survey was conducted in Yangshuo County, Guangxi Province, starting on May 18, 2020, over a 4-month period, and a total of 221 valid questionnaires were collected, with an effective collection rate of 93.644%.

In this study, 63 valid questionnaires were collected through the Internet and random distribution on the spot. Owing to the slow speed and difficulty of collecting questionnaires, this was combined with network-issued questionnaires by the screening of relevant network groups, which provided access to eligible subjects. First, questionnaires were sent out *via* WeChat, with homestay operators setting up WeChat groups and friends sharing the questionnaire information. The specific operation was as follows: the researchers used Ctrip to find the target residential accommodations and get in touch with the operators. The researchers then asked for the operators’ support and cooperation, and the homestay operators pulled former and current visitors into the same WeChat group to help forward the questionnaire. Second, the QQ Group distributed questionnaires, which were first sent directly to the QQ friends who met the identity of the surveyed users to fill in the form. Second, after searching the relevant QQ groups (Guilin mutual aid travel group and Guangxi Guilin Travel Group) and joining them, the QQ Group released the questionnaire link, asking for respondents with Yangshuo residential experience to help fill out the questionnaire. The pertinence of the Network Questionnaire ensures its authenticity and reliability, as well as improving the collection speed and yielding 158 valid questionnaires.

## Results and analysis

### Demographic characteristics of the sample

The statistical results for the demographic characteristics of respondents from the valid questionnaires are shown in Table 2.

Women accounted for 66.5% of the respondents (men, 33.5%). It appeared that women prefer to experience a homestay, pursue a specific lifestyle, and experience a different lifestyle. More than 90% of the respondents were in the range of 18–40 years old, and 80% of the respondents were 18–25 years old. Homestay consumers appeared among all of the monthly income groups, and we can see that the choice of homestay hotels at tourist destinations has become increasingly common: there are homestay tourists across all walks of life, which shows that homestays have a wide appeal for all sectors of society.

### Analysis of importance and performance

To ensure the reliability and consistency of the survey data, the Cronbach’s alpha coefficient was used to test the reliability of the questionnaire in SPSS (Oh, 2001; Bacon, 2003; Asif et al., 2019a,b); the Cronbach’s alpha coefficient for pre-consumption expectations is 0.980, the Cronbach’s alpha coefficient for post-consumption satisfaction is 0.983, and the total reliability of the questionnaire designed in this study is 0.986. According to the Reliability Index, a value of 0.8 or above indicates that the reliability of the questionnaire and its data are high; here, the values are above 0.9, which indicates that the information collected from this questionnaire is true and reliable (Wang et al., 2012; Jin and Park, 2019).

The importance and performance values of the four primary indicators (Table 4) were obtained by averaging the importance (expectation) and performance (satisfaction) values of the secondary indicators (Table 5). The entire performance of expectation value and degree of satisfaction follows the degree of satisfaction with bed and breakfast service > price cognition value satisfaction degree > function value satisfaction degree > emotion value satisfaction degree. The expectation value of the four first-class indexes is higher than the satisfaction value, while the expectation value of the homestay service is the highest, the satisfaction degree is the highest, and the affective value is the lowest for the tourists to choose a homestay. The expectation value of the first index appears to be positively correlated with the degree of satisfaction. The difference between the expectation value of the functional value and degree of satisfaction is the largest, which shows that there is a big gap between the actual situation of the homestay house and tourists’ expectations.

**TABLE 4:** Respondent demographic characteristics.

	Demographic attribute	Frequency/times	Percentage/%	Effective percentage/%	Cumulative percentage/%
Gender	Male	74	33.5	33.5	33.5
	Female	147	66.5	66.5	100.0
	Total	221	100.0	100.0	—
Age	Under 18	10	4.5	4.5	4.5
	18–25	183	82.8	82.8	87.3
	26–40	23	10.4	10.4	97.7
	41–65	5	2.3	2.3	100.0
	Over 65	0	0	0	100.0
	Total	221	100.0	100.0	—
Monthly income (RMB)	Under 1,500	115	52.0	52.0	52.0
	1,500–3,500	50	22.6	22.6	74.6
	3,501–5,000	32	14.5	14.5	89.1
	5,000 and up	24	10.9	10.9	100.0
	Total	221	100.0	100.0	—
Occupation	Staff of government agencies	7	3.2	3.2	3.2
	Staff of enterprises and institutions	31	14.0	14.0	17.2
	Self-employed	5	2.7	2.7	19.9
	Freelancer	19	8.6	8.6	28.5
	Student	139	62.9	62.9	91.4
	In the news	19	8.6	8.6	100.0
	Total	221	100.0	100.0	—

**TABLE 5:** Importance–performance analysis of the primary indicators.

Primary index	Expected value before consumption	Post-consumer satisfaction	Difference	If satisfied
A. Functional value satisfaction	3.770	3.466	−0.304	No
B. Satisfaction degree of service value of homestay	3.857	3.561	−0.296	No
C. Emotional value satisfaction	3.650	3.410	−0.240	No
D. Price perception, value satisfaction	3.801	3.511	−0.290	No
Composite situation	3.746	3.474	−0.272	No

Looking at the expected values of the secondary indicators (Table 6), the choice of the rural homestay is focused on the stability of the hot water supply, the cleanliness and tidiness of the toilet, the sound insulation of the room, the air conditioning, and the available service. Expected values at the bottom of the index ranking are provision of shuttle service, room area, and downstairs courtyard environment.

**TABLE 6:** Importance–performance analysis of the secondary indicators.

Indicator system	Average expected value before consumption	Sort by degree of expectation	Average satisfaction after consumption	Ranking of satisfaction	Difference value	*T*-value value	Probability of double tail significance (*p*)
A1. External traffic in the homestay	3.738	15	3.511	9	–0.227	3.539	0.000
A2. The homestay is close to the scenic spot	3.715	17	3.443	15	–0.272	4.301	0.000
A3. The homestay room is large	3.511	20	3.425	16	–0.086	1.280	0.202
A4. Room decoration style	3.824	8	3.475	12	–0.349	5.513	0.000
A5. The sanitary condition of the bed and breakfast room is good	3.860	5	3.466	13	–0.394	5.896	0.000
A6. The bed is comfortable	3.833	7	3.561	4	–0.272	4.324	0.000
A7. The bathroom is clean and tidy	3.928	2	3.529	5	–0.399	6.527	0.000
A8. The homestay hot water supply stable	3.955	1	3.615	1	–0.340	5.271	0.000
A9. The room air-conditioning is in normal use and no noise	3.810	10	3.511	7	–0.299	4.607	0.000
A10. The Wi-Fi in the room is fast	3.756	14	3.498	11	–0.258	4.464	0.000
A11. The room has a balcony	3.606	18	3.326	19	–0.280	3.908	0.000
A12. The room is well-insulated	3.887	4	3.394	18	–0.493	7.121	0.000
A13. Courtyard environment	3.529	19	3.321	20	–0.208	2.855	0.005
A14. Overall residential decoration style	3.778	12	3.452	14	–0.326	5.048	0.000
B1. Normal charges for bed and breakfast service	3.846	6	3.570	3	–0.276	4.239	0.000
B2. The service attitude of the accommodation staff (warm, considerate, and patient)	3.910	3	3.588	2	–0.322	5.083	0.000
B3. Room service for bed and breakfast (thoughtful, fast)	3.814	9	3.525	6	–0.289	4.714	0.000
C1. Homestay hosts provide guided tour services (professional, detailed, efficient, and considerate)	3.765	13	3.498	10	–0.267	4.525	0.000
C2. The homestay offers local food	3.719	16	3.416	17	–0.303	4.584	0.000
C3. Homestay provides shuttle service, pick up, and drop off station	3.466	21	3.317	21	–0.149	2.082	0.038
D1. Homestay is cost effective	3.801	11	3.511	8	–0.290	4.059	0.000

In terms of satisfaction with the secondary indicators (Table 6), the top 5 of the 21 indicators were the stable supply of hot water, the service attitude of the staff (warm, considerate, and patient), the normal charge for the service, the comfort of the bed, and the cleanliness and tidiness of the toilet. The top five are all in the functional value satisfaction and homestay service value satisfaction categories. The lowest degree of satisfaction was found for provision of a pick-up and drop-off car, station pick-up services, and the residential downstairs courtyard environment.

On the whole, the expectation value and the degree of satisfaction all rank in the top five of second-class index for the toilet being clean and tidy (7), the bed and breakfast hot water supply being stable (8), and the bed and breakfast service personnel’s service attitude (warm, sweet, patient) (16), with two items from the functional value degree of satisfaction belonging to the first-class index and the residential service value satisfaction degree. All 21 measures of satisfaction were below pre-consumption expectations, with the difference between expectation of and satisfaction with room sound insulation (12) reaching above 0.45, which indicates a big difference between expectation and actual satisfaction.

### Importance–performance analysis

**FIGURE 1:**
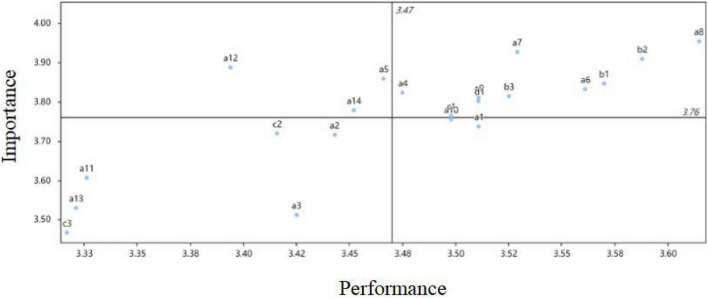
Importance–performance analysis of tourist satisfaction.

The data show that the average value of rural tourists’ pre-consumption expectation is 3.764, and the average value of the post-consumption satisfaction is 3.474. The IPA quadrant graph of the *g* satisfaction factor index is based on the general mean of expectation and the general mean of satisfaction. The horizontal axis of the IPA graph is post-consumption satisfaction, and the vertical axis is the pre-consumption expectation (Figure 1).

#### First quadrant (dominant region)

The first IPA quadrant is usually called the dominant index region, and the factor index is characterized by high expectations and satisfaction. As can be seen from Figure 1, there are 10 indicators in the dominant region, corresponding to room decoration style (A4), the comfort of the bed (A6), the cleanliness and tidiness of the toilet (A7), the stable supply of hot water (A8), the normal use and no noise of the room air-conditioning (A9), the normal charge for the room service (B1), the service attitude (warm, considerate, patient) (B2), the room service of the bed is very thoughtful and fast (B3), the host provides tour guide service (professional, detailed, efficient, and considerate) (C1), and the price of the bed is economical (D1). Of the 21 elements, these 10 aspects are in the dominant quadrant, so the rural homestay operators should pay special attention to them. It is also worth noting that, for these 10 indicators, the after-consumption satisfaction is higher but lower than pre-consumption expectations, so these 10 aspects should be the focus for improvements to enhance customer satisfaction.

#### Second quadrant (improvement area)

The second quadrant is often referred to as the key improvement (Pia) indicator area. The indicators located in this quadrant have a higher level of expectation but a lower post-consumption satisfaction, and the indicators in this quadrant area should be the focus for improvement among rural homestay operators. As can be seen from Figure 1, there are three indicators in this quadrant: sanitary condition (A5), sound insulation effect (A12), and the overall decoration style (A14). The *t*-values and two tails of these three indexes were found by SPSS Data Software as follows: good sanitary condition (A5) (*t* = 5.896, *p* = 0.000 < 0.05); sound insulation effect (A12) (*t* = 7.121, *p* = 0.000 < 0.05); and decoration style (A14) (*t* = 5.048, *p* = 0.000 < 0.05). All three indexes are lower than the pre-consumption expectation, with significance at the 5% level. This indicates that there is a significant difference between the pre-consumption expectation and the post-consumption satisfaction related to the perceived value of tourists. Substantial improvement is needed for these three indicators, which should be a focus for the development of rural residential accommodation. Both the government and the operators should increase efforts to solve the problems related to these indicators to enhance tourist satisfaction.

#### Third quadrant (opportunity area)

The third quadrant is called the opportunity index region, and items in this quadrant are characterized by low expectation and low satisfaction. As can be seen in Figure 1, there are six indicators in this region: distance from the scenic spot (A2), the large size of the room (A3), room with a balcony (A11), the downstairs courtyard environment (A13), local food characteristics (C2), and provision of shuttle or station shuttle service (C3). Factor indicators located in this area are generally considered as elements that cannot be (or are not worth being) improved because of the low expectations and satisfaction. According to the *t*-test results of the paired samples, the *p*-values for pre-consumption expectation and post-consumption satisfaction are less than 0.05 for the six indexes, except for Index 3, and the pre-consumption expectation value is larger than the post-consumption satisfaction mean value. Items 2, 11, 13, and 19 can therefore be considered sub-key improvement projects.

#### Fourth quadrant (maintenance area)

The fourth quadrant is called the maintenance index area because of its low expectation and high satisfaction. According to Figure 1, external traffic (A1) and fast Wi-Fi (A10) in the room are located in this area. The double-tail significance probability of these two indexes is equal to 0, and there appears to be a strong correlation between expectation and satisfaction, although it is the index of low expectation and high satisfaction.

## Conclusion

### Results

Homestay facilities and equipment are not perfect. The sanitary conditions, the sound insulation effect, and the entire decoration style are the three aspects for which tourists have high expectations but low satisfaction. These are key areas for improvement. Homestays lack special service development. Through the above analysis, it is clear that the opportunity to eat native special food suffers from the low expectation/low satisfaction phenomenon among target visitors, so this needs key promotion. The construction of basic roads for residential quarters still needs to be optimized. The distance from scenic spots to the homestay yields both low expectation and satisfaction. On a more positive note, the homestay service staff’s service attitude (warm, thoughtful, patient) and room service are very thoughtful and fast, yielding both high expectations and high satisfaction, and this needs to be maintained.

### Suggestions

#### Improvement of homestay facilities and environment

According to the actual circumstances of the room and the design position, the room should be soundproofed to reduce the influence of noise on the customer’s rest and improve customer satisfaction. It is also necessary to improve the standard of room hygiene. For this, it may be necessary to hire professional health service personnel, rather than choosing the cheapest cleaners to save costs. Homestays should develop a set of health service standards to ensure room hygiene. The bedclothes and sheets should be regularly disinfected and replaced to ensure the most hygienic and comfortable service for customers. During the construction of homestay rooms, appropriate steps should be taken to increase the area and strengthen room lighting, while homestays can also increase their courtyard area to provide entertainment for customers and enhance customer satisfaction.

#### Development of special services for homestays

It is necessary to strengthen the catering part of the homestay and incorporate local specialties into the menu, so that customers can enjoy the most local flavor in during the homestay experience, and reduce customers venturing to the street to eat local food. In the future development of homestays, the service of additional pick-up at the station can be increased, which would also shorten the time it takes to arrive at the homestay and thus improve the customer’s experience.

#### Optimization of basic road construction to support homestay destinations

Although external traffic had lower expectations and higher satisfaction in the data results, it is not necessary for homestays in Yangshuo to develop naturally. Contacts with local homestay operators in Yangshuo during a Ctrip internship revealed that, due to the government’s efforts to repair roads to scenic spots and restrict the entry of non-local vehicles, the occupancy rate of many homestays has been affected, causing many businesses to complain. It is hoped that the relevant government departments will increase the efficiency of the engineering team during the road construction period and ensure quality and efficiency, which would reduce the impact of the road on homestays, allowing them to improve their occupancy rates and increase profits.

#### Quality improvement of homestay operators

Homestay operators should study the relevant material for running a bed and breakfast and improve their knowledge and self-cultivation, so they can give the customers the most comfortable feeling in terms of amenities and style, as well as enhance the customer’s experience. Homestay operators regularly learn the relevant esthetic collocation knowledge in terms of overall decoration and homestay items, allowing them to draw on their best judgment to enhance customer satisfaction in terms of decoration style. They can also solicit feedback from customers to enhance the homestay experience. It is also necessary to strengthen the personality training of homestay workers and encourage them to have more in-depth exchanges with customers, including small talk about local scenic spots and in-depth communication about local culture. This will strengthen the creation of a homestay general atmosphere and improve customer satisfaction.

## Discussion

This study uses IPA analysis to analyze the tourist satisfaction of rural homestays and proposes improvement methods and suggestions in four major aspects: facilities and environment of homestays, development of special services, optimization of basic road construction, and quality improvement of homestay operators, in order to improve the shortcomings of rural homestays and provide a new research method for the study of tourist perception value of homestays. This study provides a new research methodology. It also aims to increase the income of rural homestay operators and to make rural homestays sustainable. The study also aims to increase the income of rural homestay operators and make the sustainable development of rural homestays, which has important practical and theoretical significance for the sustainable development of rural homestays.

## Research limitation and further research

The evaluation index system assessing the degree of satisfaction with tourists to rural areas constructed in this study needs further verification and improvement in future research, as there are some deficiencies in it. It is hoped that, in a following study, the sample data will be expanded and the occupational percentages of the subjects will be averaged to ensure the validity of the data and the authenticity of the results. The object of this study was only Yangshuo, which is a tourist destination within the three provinces of southwest Yunnan, Guangxi, and Guizhou. Increasing the sample size would make the data analysis more representative, and the analysis results can be used to promote the healthy development of rural homestays.

## Data availability statement

The original contributions presented in the study are included in the article/supplementary material, further inquiries can be directed to the corresponding author.

## Ethics statement

Ethical review and approval was not required for the study on human participants in accordance with the local legislation and institutional requirements. Written informed consent was obtained from all participants for their participation in this study.

## Author contributions

XZ: conceptualization, methodology, and funding acquisition. PM: software. YW and PM: formal analysis. YW: investigation and data curation. XZ and PM: writing—original draft preparation. XZ and YW: writing—review and editing. All authors have read and agreed to the published version of the manuscript.
